# Role and Risk Factors of Glycosylated Hemoglobin Levels in Early Disease Screening

**DOI:** 10.1155/2021/6626587

**Published:** 2021-04-08

**Authors:** Guanqun Chao, Yue Zhu, Liying Chen

**Affiliations:** Department of General Practice, Sir Run Run Shaw Hospital, Zhejiang University, China

## Abstract

**Objective:**

To clarify the correlation among glycosylated hemoglobin (HbA1c), gender, age, fatty liver, and biochemical indicators through the analysis of big database, and to further investigate the risk factors affecting HbA1c, so as to lay a foundation for the study of HbA1c-related diseases and disease management.

**Methods:**

People who have been examined in Health Promotion Center from July 2018 to June 2019 were selected as the research objects. All data were analyzed using the Windows R software (version 3.5.1). Detailed medical history inquiry, laboratory examination, and B-ultrasound examination were carried out for the selected sample population. We determined the sample population according to the inclusion and exclusion criteria and, then, further grouped and analyzed the data. *T*-test or Mann–Whitney *U* test was used for continuous variable comparison, and chi-square test was used to compare categorical variables. Logistic regression analysis was used to analyze the risk factors of HbA1c.

**Results:**

A total of 23,933 subjects were included in this study. The HbA1c level of men was significantly higher than that of women, the HbA1c level of the group with diabetes was higher than that of the group with no diabetes, and the HbA1c level of the group with fatty liver was higher than that of the group with no fatty liver. In the group with no diabetes, the HbA1c level increased with weight gain. Age, gender (male), fatty liver, waist circumference, systolic blood pressure (SBP), triglyceride (TG), blood urea nitrogen (BUN), free thyroxine (FT4), and red blood cell (RBC) were the risk factors for elevated HbA1c level, while high-density lipoprotein cholesterol (HDL-C), uric acid (UA), creatinine (Cr), free triiodothyronine (FT3), and hemoglobin were protective factors.

**Conclusion:**

Blood glucose, age, weight, gender, fatty liver, blood lipids, and UA are related to the increase of HbA1c level. HbA1c is related to many metabolic indexes and may be used as a marker for early detection of chronic diseases.

## 1. Introduction

With the development of economy, diabetes is affecting relatively wide portions of the world population, especially in developed countries, and the complications of diabetes are rising in younger people and earlier [1]. Obesity is an important risk factor of type 2 diabetes. With the improvement of living standards, the number of obese people increases gradually, causing the gradually mounting number of patients with diabetes, as well as the number of younger patients in particular [2]. There are nearly 180 million patients with diabetes in the world [3]. Glycosylated hemoglobin (HbA1c) plays an important role in the management of diabetes, which has been regarded as one of the most important research progress in the treatment of diabetes for decades [4]. HbA1c has been used as an index for clinical diagnosis of diabetes in foreign countries. However, it is found that the threshold value of HbA1c is not suitable for young people, especially for Chinese people [5]. As we know, HbA1c reflects the average blood glucose of about three months, which is closely related to the complications of diabetes [6].

HbA1c is widely used in blood glucose management of gestational diabetes mellitus in addition to the clinical significance of HbA1c for type 1 and type 2 diabetes mellitus [7]. HbA1c is affected by many other factors. The study shows that HbA1c is related to people's lifestyle, such as strenuous exercise and carbohydrate control [8]. It is well proved that HbA1c correlates with the life cycle of red blood cells. Recent studies suggest that the results of HbA1c are affected by iron deficiency anemia and related with the degree of anemia [9]. However, another study finds that iron supplementation during pregnancy does not affect the level of HbA1c and has no clinical effect on the final interpretation of the results in patients with no anemia or mild anemia [10]. High HbA1c and fasting blood glucose levels are believed to significantly alter the relationship between HbA1c, glucose, and age [11]. On the other hand, HbA1c is also considered to be a risk factor for cardiovascular disease in patients [12]. At present, the research on HbA1c mostly focuses on the control of diabetes and the standard of detection. Many studies believe that HbA1c is related to many factors, but the results are controversial. Therefore, we designed this study, hoping to clarify the correlation between HbA1c and other factors through the analysis of big data, such as gender, age, fatty liver, and biochemical indicators, and to further investigate the risk factors affecting HbA1c, in a bid to lay a foundation for the study of HbA1c-related diseases and chronic disease management.

## 2. Materials and Methods

### 2.1. Data and Methods

This study is a retrospective analysis. People who have been examined in the Health Promotion Center of Sir Run Run Shaw Hospital affiliated with Zhejiang University in Hangzhou in China from July 2018 to June 2019 were selected as the research objects.

Inclusive criteria are the following: (1) detailed medical history collection and (2) completion of examination items in physical examination center.

Exclusion criteria are the following: (1) less than 18 years old, (2) pregnancy or lactation, (3) patients with malignant tumor, (4) patients with liver cirrhosis, (5) patients with chronic kidney disease, (6) patients with heart disease, and (7) patients with autoimmune diseases.

### 2.2. Clinical and Laboratory Assessments

The medical history was collected by senior general practitioners. The contents of medical history collection include current medical history (existing symptoms, drugs being taken, and so on), past history (operation history, blood transfusion history, past disease history, and so on), and family history. Weight, height, waist circumference, and blood pressure were completed by trained nurses. Body mass index (BMI) was calculated as body weight (kg)/height squared (m^2^). Serum fasting blood glucose (FBG), total cholesterol (TC), triglyceride (TG), uric acid (UA), aminotransferase (ALT), high-density lipoprotein cholesterol (HDL-C), blood urea nitrogen (BUN), low-density lipoprotein cholesterol (LDL-C), aspartate aminotransferase (AST), and creatinine (Cr) were measured using a Hitachi 7600 clinical analyzer (Hitachi, Tokyo, Japan). Thyroid function including thyroid-stimulating hormone (TSH), free thyroxine (FT4), free triiodothyronine (FT3), thyroid globulin antibody (Tg-Ab), and thyroid peroxidase antibody (TPO-Ab) were quantified by chemiluminescent enzyme immunoassays (ICMA; Abbott, Chicago, IL, USA). The blood sample for laboratory tests was drawn from individuals after 8 h or more of fasting.

### 2.3. Diagnostic Criteria

Diagnosis of patients with diabetes: diabetes has been clinically diagnosed with blood glucose more than 7.0 mmol/I [13] or in the case of oral hypoglycemic drugs assumption. Fatty liver was diagnosed according to the results of B ultrasound. According to BMI, it was divided into underweight (BMI < 18.5 kg/m^2^) group, overweight (BMI ≥ 24 kg/m^2^) group, and normal weight (18.5 kg/m^2^≦BMI < 24 kg/m^2^) group.

### 2.4. Statistical Analysis

All data were analyzed using the Windows R software (version 3.5.1). This study is a retrospective study using secondary analysis. *T*-test or Mann–Whitney *U* test was used for continuous variable comparison, and chi-square test was used to compare categorical variables. The *t*/*χ*^2^ tests were used to compare the differences between the demographics and characteristics of two groups. The univariate analysis was used to compare the differences between the demographics and characteristics of three or more groups. Multiple linear regression was used to compare the relationship between HbA1c and gender, age, weight, and other biochemical factors. A multivariate logistic regression model was used to evaluate associated factors with the elevation of HbA1c. In this model, whether HbA1c elevated over 6.5% was defined as the dependent variables, and factors such as age, sex, fatty liver condition, waist circumference, and systolic blood pressure were defined as the independent variables. The mean of continuous data is expressed as mean ± SD. *p* < 0.05 was statistically significant.

## 3. Results

### 3.1. Comparison of Baseline Data between Men and Women

**Figure 1:**
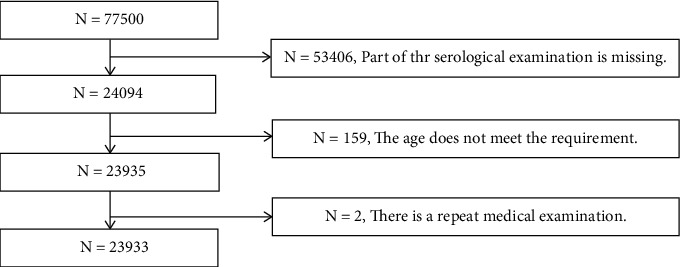
Flow diagram.

Finally, a total of 23,933 subjects were included in this study, including 13,566 men and 10,367 women (Figure 1). There were significant differences between men and women in age, BMI, waist circumference, blood pressure, FBG, blood lipid, thyroid function, liver, and kidney function. Age, BMI, waist circumference, blood pressure, RBC count, and hemoglobin in men were higher than those in women; FBG in men was higher than those in women, glycosylation in men was higher than those in women; blood lipids in men were higher than those in women; liver enzymes in men were higher than those in women; UA in men was higher than those in women; CR and BUN in men were higher than those in women; thyroxine in men was higher than those in women; thyrotropin in women was higher than those in men; thyroid hormone in women was higher than that of men. The antibody of women was higher than that of men (Table 1).

In the men group, HBA1c of the three groups with different BMI levels were different and showed an upward trend with the increase of BMI (*p* ≤ 0.01). In the men group, HBA1c of the group with no fatty liver was different from that of the group with fatty liver, and HBA1c of the group with fatty liver was higher (*p* ≤ 0.01). In the men group, the proportion of HbA1c increased in the three groups with different BMI levels was different (*p* ≤ 0.01). In the men group, the proportion of increased HbA1c in the group with fatty liver was higher (*p* ≤ 0.01). In the women group, glycosylated hemoglobin of the three groups with different BMI levels was different and showed an upward trend with the increase of BMI (*p* ≤ 0.01). In the women group, HbA1c was higher in the group with fatty liver (*p* ≤ 0.01). In the women group, the proportion of HbA1c increased in the three groups with different BMI levels was different (*p* ≤ 0.01). In the men group, the proportion of HbA1c increased in the group with fatty liver was higher (*p* ≤ 0.01).

### 3.2. Comparison of Baseline Data between Group with Diabetes and Group with No Diabetes

The included samples were divided into group with diabetes and group with no diabetes (Table 2). There were significant differences in age, BMI, waist circumference, blood pressure, FBG, blood lipid, thyroid function, and renal function between group with diabetes and group with no diabetes, but there were no significant differences in AST and TGAb. The age, BMI, waist circumference, blood pressure, RBC count, hemoglobin, fasting FBG, glycosylation, TG, LDL, ALT, UA, CR, and BUN of group with diabetes were lower than those of group with no diabetes. The levels of TC, HDL, FT3, TSH, TGAb, and TPOAb in group with diabetes were higher than those in group with no diabetes.

In the group with no diabetes, the HbA1c of the three groups with different BMI levels were different and showed an upward trend (*p* ≤ 0.01). In the group with no diabetes, HbA1c of group with fatty liver was higher (*p* ≤ 0.01). In the group with no diabetes, the proportion of HbA1c increased was different among the three groups with different BMI levels (*p* ≤ 0.01). In the group with no diabetes, the proportion of HbA1c increased in the group with fatty liver was higher (*p* ≤ 0.01). In the group with diabetes, HbA1c of the three groups with different BMI levels was the same (*p* = 0.8). In the group with diabetes, HbA1c of group with fatty liver was higher (*p* ≤ 0.01). In the group with diabetes, the proportion of people with elevated HbA1c was the same among the three groups with different BMI levels (*p* = 0.341). In the group with no diabetes, the proportion of HbA1c increased in the group with fatty liver was higher (*p* ≤ 0.01).

### 3.3. Correlation between HbA1c and Body Weight

According to BMI, the samples of group with diabetes and group with no diabetes were divided into three groups: group with underweight, group with normal weight, and group with overweight. Analysis of variance showed that HbA1c increased with weight gain in group with no diabetes, but not in group with diabetes. (Table 3).

### 3.4. Correlation between HbA1c and Fatty Liver

Fatty liver was diagnosed according to the results of B-ultrasound. Group with diabetes and group with no diabetes were divided into two groups: fatty liver and no fatty liver. In the group with no diabetes, there were 7,659 patients in the group with fatty liver and 15,061 people in the group with no fatty liver. The HbA1c in the group with fatty liver was significantly higher than that in the group with no fatty liver (5.58 ± 0.72 vs. 5.29 ± 0.44). In the group with diabetes, there were 763 patients in the group with fatty liver and 450 people in the group with no fatty liver. In the group with diabetes, the HbA1c in the group with fatty liver was significantly higher than that in the group with no fatty liver (7.20 ± 1.24 vs. 6.97 ± 1.45).

### 3.5. Linear Correlation Analysis of HbA1c

The results of multiple linear regression analysis showed that HbA1c had linear correlation with age, gender (men), fatty liver, SBP, RBC, hemoglobin, TC, HDLC, LDLC, UA, BUN, FT3, and FT4.

### 3.6. The Correlation between the Increase of HbA1c (>6.5%) and Diabetes Mellitus, Fatty Liver, and Body Weight

The results of chi-square analysis showed that HbA1c increased more in group with diabetes (792/1213) than in group with no diabetes(653/22720) (*p* < 0.05).

Fisher's exact test showed that in the group with no diabetes, there was difference in the proportion of patients with elevated HbA1c in the group with underweight (3/683), the group with normal weight (135/11172), and the group with overweight (515/10865). The proportion of patients with high HbA1c increased with the increase of body weight. There was no difference in the proportion of patients with elevated HbA1c in group with diabetes (group with underweight (5/9), group with normal weight (245/390), and group with overweight (542/814)).

Chi-square analysis showed that in the group with no diabetes, the proportion of HbA1c in the group with fatty liver (513/7659) was significantly higher than that in the group with no fatty liver (140/15061). In group with diabetes, the proportion of HbA1c increased significantly in the group with fatty liver (529/763) than that in the group with no fatty liver (263/450).

### 3.7. Logistic Regression Analysis of Risk Factors for Elevated HbA1c

Finally, the logistic model was statistically significant. In the model, age, gender (men), fatty liver, waist circumference, SBP, RBC number, hemoglobin, blood lipid (TG, HDLC), UA, BUN, Cr, FT3, and FT4 were statistically significant. The results showed that the risk of elevated HbA1c increased by 6.7% for each year of age increase. The risk of elevated HbA1c was 2.45 times higher in men than in women. Compared with patients without fatty liver, patients with fatty liver had a two-fold increased risk of HbA1c. For every 1 cm increase in waist circumference, the risk of elevated HbA1c increased by 5.7%. For each unit of SBP increase, the risk of elevated HbA1c increased by 0.9%. When the number of RBC increased by 1 × 10^12^, the risk of elevated HbA1c increased by 53.9%. For every 1 g/L increase in hemoglobin, the risk of elevated HbA1c decreased by 0.9%. For each unit of TG increase, the risk of elevated HbA1c increased by 10.2%. For each additional unit of HDLC, the risk of elevated HbA1c decreased by 48.0%. For each unit of UA increase, the risk of elevated HbA1c decreased by 0.5%. For each unit of BUN increase, the risk of elevated HbA1c increased by 26.5%. For each unit of Cr increase, the risk of elevated HbA1c decreased by 3.4%. When FT3 increased by each unit, the risk of elevated HbA1c decreased by 58.0%. The risk of elevated HbA1c increased by 5.758 times for each unit of FT4 (Table 4).

## 4. Discussion

The results indicate that differences exist in baseline and biochemical indexes between men and women, and the HbA1c of men is significantly higher than that of women. A recent study suggests that the HbA1c of men is higher than that of women in the age of 20-59 [14], while the average age of the sample included in this study is 47.94 ± 10.32, so the results are consistent with it. Previous studies [15] have also suggested that age and gender should be taken into account in the diagnosis of diabetes with HbA1c, which shows that there is a correlation between gender and HbA1c. Similarly, this study finds that HbA1c is significantly higher in patients with diabetes than patients with no diabetes. For patients with no diabetes, HbA1c increased with the increase of body weight, while in group with diabetes, there was no significant correlation between weight and HbA1c. Compared with patients with no fatty liver, the HbA1c of patients with fatty liver is higher regardless of whether they have diabetes or not. Studies suggest that HbA1c has low sensitivity and high specificity in the diagnosis of diabetes and prediabetes, which varies with age and race [16]. Therefore, HbA1c may be affected by many factors.

HbA1c level is recommended by the American Diabetes Association (ADA) to be used as an index for the diagnosis of diabetes [17], and it can reflect blood glucose level of 2-3 months. It is suggested that HbA1c can be used in the diagnosis of diabetes [18]. HbA1c is becoming more and more popular among primary care providers because it has many practical advantages, including convenient sampling, suitable as an indicator of chronic abnormal blood glucose, low individual variation, and favorable laboratory standardization [19]. In 2010, ADA proposed that HbA1c value of 5.7%-6.4% can be diagnosed as prediabetes and HbA1c ≥6.5% can be diagnosed as diabetes [13], which is a criteria for diagnosis recommended by ADA. In our physically examined population, patients with diabetes included drug users and nondrug users, and HbA1c, as an important indicator of blood glucose management, can show that patients with diabetes in physically examined population do not control blood glucose well.

A recent study suggested that fatty liver is associated with insulin resistance, and HbA1c in group with fatty liver is significantly higher than that in group with no fatty liver [20], which is consistent with our results. With regard to metabolic risk, nonalcoholic fatty liver disease (NAFLD) is a powerful and highly prevalent predictor of type 2 diabetes [21]. A domestic study suggests that serum HbA1c level is associated with NAFLD, and the increase of serum HbA1c level is an independent risk factor for NAFLD in Chinese elderly [22]. Therefore, patients with fatty liver need to be alert to diabetes, and conversely, according to our research, patients with fatty liver have elevated HbA1c. Thus, for those with elevated HbA1c found in physical examination, doctors should not only pay attention to diabetes but also pay attention to fatty liver. The researchers suggest that NAFLD may transform into liver cancer, which is an important risk factor for it. Therefore, the correlation between HbA1c and fatty liver deserves the attention of doctors. Another study finds that HbA1c is significantly related to BMI and waist circumference [23], which is consistent with the results of our study on the increase of HbA1c with weight gain in patients with no diabetes, but it is inconsistent with the results of group with diabetes, which may be due to the small sample size.

At present, the research on the relationship between age and HbA1c is still limited. Studies have confirmed that HbA1c increases with age in individuals with no diabetes [24]. Some studies have found that the diagnostic efficiency of HbA1c for diabetes decreases with age, which is caused by the decrease of RBC count with age [25]. For adolescents and children, the study finds that HbA1c level decreased every 100 percentile from 2 to 4 years old, increased gradually before puberty, reached the peak at 12 to 14 years old, and then dropped to the lowest level in the third decade [26]. The results of this study suggest that the risk of elevated HbA1c increases by 6.7% for each year of age increase. In view of the correlation between gender, fatty liver, waist circumference, and HbA1c, this study also finds that men, fatty liver, and waist circumference increase the risk of elevated HbA1c. In the study of type 1 diabetes, it is found that HbA1c, night heart rate, and height were related to blood pressure changes [27]. Studies also suggest that elevated blood pressure has contributed to the increase in incidence rate of stroke, which is related to elevated blood glucose level represented by HbA1c [28]. This study finds that for each unit of SBP increase, the risk of elevated HbA1c increased by 0.9%. Iron deficiency anemia can affect the homeostasis of glucose and the control of blood glucose [29]. The mechanism is that iron deficiency accelerates glycosylation by changing the structure of hemoglobin and inducing peroxidation [30]. It can be seen that hemoglobin is related to HbA1c.

A cross-sectional study shows that HbA1c is significantly correlated with serum TC, TG, and HDLC levels in patients with diabetes, but not with LDLC [31]. One study finds that TC and abnormal LDL-C values of patients with HbA1c ≥7.0% were significantly higher than those of patients with HbA1c <7.0% [32]. At the same time, studies on patients with type 2 diabetes have found that serum uric acid concentration is associated with microalbuminuria and glycosylated hemoglobin [33]. These results are consistent with our results, and our study further found that the risk of elevated HbA1c increased by 10.2% for each unit of TG and decreased by 48.0% for each unit of HDLC. UA is considered to be related to HbA1c level [34]. Our study suggests that for each unit of UA increase, the risk of elevated HbA1c decreases by 0.5%. Similarly, a recent study finds that UA is negatively correlated with HbA1c in newly diagnosed patients with diabetes but is associated with hyperinsulinemia. If insulin is used, UA and HbA1c are not significantly correlated [35]. Besides, low serum creatinine is considered to be associated with an increased risk of diabetes, and screening serum creatinine levels can be used to identify high-risk groups of diabetes [36]. Our study suggests that the risk of elevated HbA1c decreases by 3.4% for each unit of Cr increase, which is consistent with the literature results. The incidence of thyroid dysfunction in patients with type 2 diabetes is higher and increased with the increase of HBA1c [37]. This may explain the correlation between thyroid hormone and HbA1c. However, we have not been able to find the research related to policy. Therefore, we hope our research can exert important significance for the early screening of diseases.

## 5. Strengths and Limitations

The advantage of our research lies in the large sample size, the samples are from the same hospital's health promotion center, and the data has the characteristics of authenticity and objectivity. However, there are still some limitations in this study: (1) the diagnosis of diabetes mellitus is only based on the medical history; (2) the included samples in the group with diabetes do not distinguish drug assumptions or not; (3) this study is a cross-sectional study, which can only explain the results of a certain level, but cannot reflect the changes in a period of time; (4) it is impossible to make a clear and detailed history of drinking; (5) the diagnosis of fatty liver is only based on the results of B ultrasound; (6) it is impossible to distinguish whether the sample population is carrying out lifestyle intervention. We hope to design prospective studies or clinical controlled trials in the future and confirm the relevant research conclusions through more data. On this basis, we will try our best to eliminate interference factors and establish clinical controlled trials to clarify the correlation between various metabolic diseases and HbA1c.

## 6. Conclusion

This study found that HbA1c levels are not only associated with diabetes but also with a variety of other metabolic factors. HbA1c is associated with body weight in population with no diabetes. The level of HbA1c in patients with fatty liver is higher than that in patients with no fatty liver. In addition, HbA1c level is linearly correlated with body weight, SBP, fatty liver, gender, age, blood lipid, thyroid hormone, UA, and renal function. Therefore, the increase of HbA1c may be related to many diseases and abnormal biochemical indexes. It is suggested that clinicians need to screen other metabolic diseases when HbA1c is abnormal in physical examination, which will be conducive to the development of clinical health management and early screening of diseases. In conclusion, HbA1c is associated with many metabolic indicators, which can be further developed as a special marker for early screening of chronic diseases.

## Figures and Tables

**Table 1:** Comparison of baseline data between men and women.

	Women *n* = 10367	Men *n* = 13566	*p*
Age	46.92 ± 10.54	47.94 ± 10.32	*p* ≤ 0.01
BMI	22.80 ± 2.99	25.03 ± 3.10	*p* ≤ 0.01
WC	77.27 ± 8.58	89.20 ± 8.52	*p* ≤ 0.01
SBP	117.44 ± 17.59	124.91 ± 15.04	*p* ≤ 0.01
DBP	69.00 ± 10.91	75.59 ± 10.70	*p* ≤ 0.01
FBG	5.14 ± 0.86	5.50 ± 1.33	*p* ≤ 0.01
RBC	4.31 ± 0.35	4.91 ± 0.40	*p* ≤ 0.01
HB	129.67 ± 11.40	152.10 ± 10.32	*p* ≤ 0.01
HBA1C	5.38 ± 0.60	5.56 ± 0.81	*p* ≤ 0.01
TG	1.31 ± 0.98	1.99 ± 1.71	*p* ≤ 0.01
TC	4.77 ± 0.93	4.83 ± 0.94	*p* ≤ 0.01
HDL	1.42 ± 0.33	1.16 ± 0.28	*p* ≤ 0.01
LDL	2.62 ± 0.73	2.74 ± 0.76	*p* ≤ 0.01
ALT	18.99 ± 15.26	31.20 ± 26.19	*p* ≤ 0.01
AST	18.58 ± 8.41	22.14 ± 13.42	*p* ≤ 0.01
UA	287.27 ± 59.84	400.56 ± 78.26	*p* ≤ 0.01
BUN	4.56 ± 1.14	5.06 ± 1.16	*p* ≤ 0.01
CR	57.79 ± 8.61	80.65 ± 13.24	*p* ≤ 0.01
FT3	2.80 ± 0.41	2.97 ± 0.37	*p* ≤ 0.01
FT4	1.00 ± 0.12	1.02 ± 0.11	*p* ≤ 0.01
TSH	2.00 ± 1.47	1.71 ± 1.44	*p* ≤ 0.01
TGAB	25.62 ± 93.61	7.73 ± 45.52	*p* ≤ 0.01
TPOAB	27.42 ± 110.39	10.29 ± 64.41	*p* ≤ 0.01

Note: BMI: body mass index; WC: waist circumference; SBP: systolic blood pressure; DBP: diastolic blood pressure; FBG: fast blood glucose; HBA1C: glycated hemoglobin; TC: total cholesterol; TG: triglycerides; LDL: low-density lipoprotein; HDL: high-density lipoprotein; ALT: alanine aminotransferase; AST: Aspartate aminotransferase; UA: serum uric acid; BUN: blood urea nitrogen; CR: creatinine; FT3: free Triiodothyronine; FT4: free thyroxine; TSH: thyroid-stimulating hormone; TGAB: thyroid peroxidase antibody; TPOAB: thyroglobulin antibody. *p* < 0.05 was considered statistically significant.

**Table 2:** Comparison of baseline data between diabetes group and nondiabetes group.

	Nondiabetes group *n* = 22720	Diabetes group *n* = 1213	*p*
Age	47.09 ± 10.36	55.04 ± 8.76	*p* ≤ 0.01
Male sex	12645 (55.7%)	921 (75.9%)	*p* ≤ 0.01
BMI	23.99 ± 3.24	25.49 ± 3.06	*p* ≤ 0.01
WC	83.68 ± 10.33	90.75 ± 9.08	*p* ≤ 0.01
SBP	121.30 ± 16.54	128.80 ± 16.24	*p* ≤ 0.01
DBP	72.58 ± 11.31	75.58 ± 10.16	*p* ≤ 0.01
FBG	5.21 ± 0.85	7.94 ± 2.46	*p* ≤ 0.01
HBA1C	5.39 ± 0.57	7.11 ± 1.33	*p* ≤ 0.01
TG	1.68 ± 1.45	2.09 ± 1.91	*p* ≤ 0.01
TC	4.81 ± 0.93	4.66 ± 1.06	*p* ≤ 0.01
HDL	1.27 ± 0.32	1.16 ± 0.29	*p* ≤ 0.01
LDL	2.69 ± 0.75	2.53 ± 0.81	*p* ≤ 0.01
ALT	25.75 ± 22.99	29.04 ± 21.86	*p* ≤ 0.01
AST	20.57 ± 11.43	20.98 ± 15.22	0.326
UA	350.97 ± 90.68	361.21 ± 84.65	*p* ≤ 0.01
BUN	4.82 ± 1.16	5.34 ± 1.41	*p* ≤ 0.01
CR	70.68 ± 16.00	72.02 ± 18.13	0.011
FT3	2.90 ± 0.40	2.81 ± 0.36	*p* ≤ 0.01
FT4	1.01 ± 0.11	1.03 ± 0.12	*p* ≤ 0.01
TSH	1.84 ± 1.48	1.71 ± 1.12	*p* ≤ 0.01
TGAB	15.75 ± 71.73	10.56 ± 56.69	*p* ≤ 0.01
TPOAB	17.94 ± 88.22	13.49 ± 78.19	0.055

Note: BMI: body mass index; WC: waist circumference; SBP: systolic blood pressure; DBP: diastolic blood pressure; FBG: fast blood glucose; HBA1C: glycated hemoglobin; TC: total cholesterol; TG: triglycerides; LDL: low-density lipoprotein; HDL: high-density lipoprotein; ALT: alanine aminotransferase; AST: aspartate aminotransferase; UA: serum uric acid; BUN: blood urea nitrogen; CR: creatinine; FT3: free triiodothyronine; FT4: free thyroxine; TSH: thyroid-stimulating hormone; TGAB: thyroid peroxidase antibody; TPOAB: thyroglobulin antibody. *p* < 0.05 was considered statistically significant.

**Table 3:** Correlation between HbA1c and body weight.

Nondiabetes group	Underweight (BMI < 18.5 kg/m^2^) *N* = 683	Normal weight (18.5 kg/m^2^≦BMI < 24 kg/m^2^) *N* = 11172	Overweight (BMI ≥ 24 kg/m^2^) *N* = 10865	*p*
Hba1c	5.17 ± 0.38	5.29 ± 0.47	5.51 ± 0.64	*p* ≤ 0.01
Diabetes group	Underweight (BMI < 18.5 kg/m^2^) *N* = 9	Normal weight (18.5 kg/m^2^≦BMI < 24 kg/m^2^) *N* = 390	Overweight (BMI ≥ 24 kg/m^2^) *N* = 814	*p*
Hba1c	7.00 ± 2.15	7.08 ± 1.45	7.13 ± 1.26	0.778

Note: BMI: body mass index; analysis of variance was used.

**Table 4:** Logistic regression analysis of risk factors for elevated HbA1c.

	Age	MALE	Fatty liver	Wc	sbp	TG	HDLC	UA
OR	1.067	3.448	3.002	1.057	1.009	1.102	0.520	0.995
*p*	≤0.01	≤0.01	≤0.01	≤0.01	≤0.01	≤0.01	≤0.01	≤0.01
	Bun	Cr	Ft3	Ft4	RBC	HB		
OR	1.265	0.966	0.420	6.758	1.539	0.991		
*p*	≤0.01	≤0.01	≤0.01	≤0.01	≤0.01	≤0.01		

Note: BMI: body mass index; WC: waist circumference; SBP: systolic blood pressure; DBP: diastolic blood pressure; FBG: fast blood glucose; HBA1C: glycated hemoglobin; TC: total cholesterol; TG: triglycerides; LDL: low-density lipoprotein; HDL: high-density lipoprotein; ALT: alanine aminotransferase; AST: aspartate aminotransferase; UA: serum uric acid; BUN: blood urea nitrogen; CR: creatinine; FT3: free triiodothyronine; FT4: free thyroxine; TSH: thyroid-stimulating hormone; TGAB: thyroid peroxidase antibody; TPOAB: thyroglobulin antibody. *p* < 0.05 was considered statistically significant. OR > 1 is risk factor; OR < 1 is protective factor.

## Data Availability

The data used to support the findings of this study are available from the corresponding author upon request.
